# Thoracic Endometriosis Masquerading As Recurrent Hemothorax

**DOI:** 10.7759/cureus.67235

**Published:** 2024-08-19

**Authors:** Kubiat E Udoh, Andikan E Udoh, Maithri Vallabhaneni, Vishal Busa, Shravani Surakanti

**Affiliations:** 1 Internal Medicine, Baton Rouge General, Baton Rouge, USA; 2 Internal Medicine, Baton Rouge General, Louisiana, USA

**Keywords:** endometrial stroma, video-assisted thoracoscopic surgery (vats), recurrent hemothorax, catamenial hemothorax, thoracic endometriosis

## Abstract

Extragenital endometriosis is not a common occurrence. Its diagnosis is often delayed, which leads to further complications and recurrent hospitalizations. In this report, we present a case of a 37-year-old African American female diagnosed with thoracic endometriosis who initially presented with a two-week duration of progressive shortness of breath. The diagnosis of this patient posed a dilemma as there was initial suspicion of ovarian cancer and Meig’s syndrome, which can have similar presentations.

## Introduction

Extragenital endometriosis is not a common occurrence, with an incidence of approximately 12% in reproductive-aged women. Thoracic endometriosis is described as a hallmark of endometriosis progression and the presence of endometrial glands and stroma within the thoracic cavity [1,2]. The thoracic cavity is the most common extragenital site of endometriosis, and clinical presentation varies. Although the most common presentation of thoracic endometriosis is pneumothorax, hemothorax has also been frequently encountered; and in most cases, it tends to be right-sided in presentation [1,3]. 

It is often related to the menstrual cycle, a phenomenon termed “catamenial,” but this does not occur in every situation, and it is frequently encountered in middle-aged women between the ages of 35 and 45 [1,3]. Multiple theories exist regarding the pathogenesis of thoracic endometriosis, but not one or the other can explain the constellation of signs and symptoms associated with it; it remains a controversial topic [1,3]. 

In this article, we will discuss hemothorax with a pattern of recurrence as a symptom of thoracic endometriosis in a young female that posed a diagnostic challenge. 

## Case presentation

A 37-year-old female with no significant history except dysmenorrhea presented to the emergency department with complaints of shortness of breath worse on exertion accompanied by a productive cough with slightly mucoid sputum and brief episodes of chest pain ongoing for two weeks prior to presentation. 

On arrival, her vitals were recorded; blood pressure was 150/96 mmHg, heart rate was 99, respiratory rate was 18, temperature was 99.3F, and oxygen saturation was 100% on room air. The physical exam was notable for absent breath sounds over the right lower and middle lung zones. Labs were obtained, and the complete blood count was remarkable for Hemoglobin (10g/dL) and mean corpuscular volume (74 fL). The cancer antigen 125 level was also obtained at 173.4 U/mL. Laboratory values of a few chemistry and hematology tests are listed, the rest of the laboratory values were unremarkable (Table 1).

**Table 1:** Laboratory Values of Pertinent Chemistry and Hematology Tests CEA: carcinoembryonic antigen, AFP: alpha-fetoprotein, CA19: cancer antigen 19-9, CA-125: cancer antigen-125, MCV: mean corpuscular volume, TIBC: total iron binding capacity, LDH: lactate dehydrogenase

Chemistry/Hematology	Laboratory Values	Reference Range
CEA	1.0	0.0-3.5 ng/mL
AFP	3.8	0.5-8.0 ng/mL
CA19-9	<2.00	2.00-37.00 U/mL
CA-125	173.4	1.5-35.0 U/mL
Hemoglobin	10	11.2-15.7 g/dL
Hematocrit	33.4	34.1-44.9%
MCV	74	79-95 fL
Platelets	412	150-400 K/uL
Iron	29	50-170 ug/dL
% Saturation	8.7	20.0-50.0%
Ferritin	30.9	8.0-252 ng/mL
TIBC	332	250-480 ug/dL
Serum LDH	166	84-246 U/L
Serum total protein	8.0	6.4-8.6 g/dL

**Figure 1:**
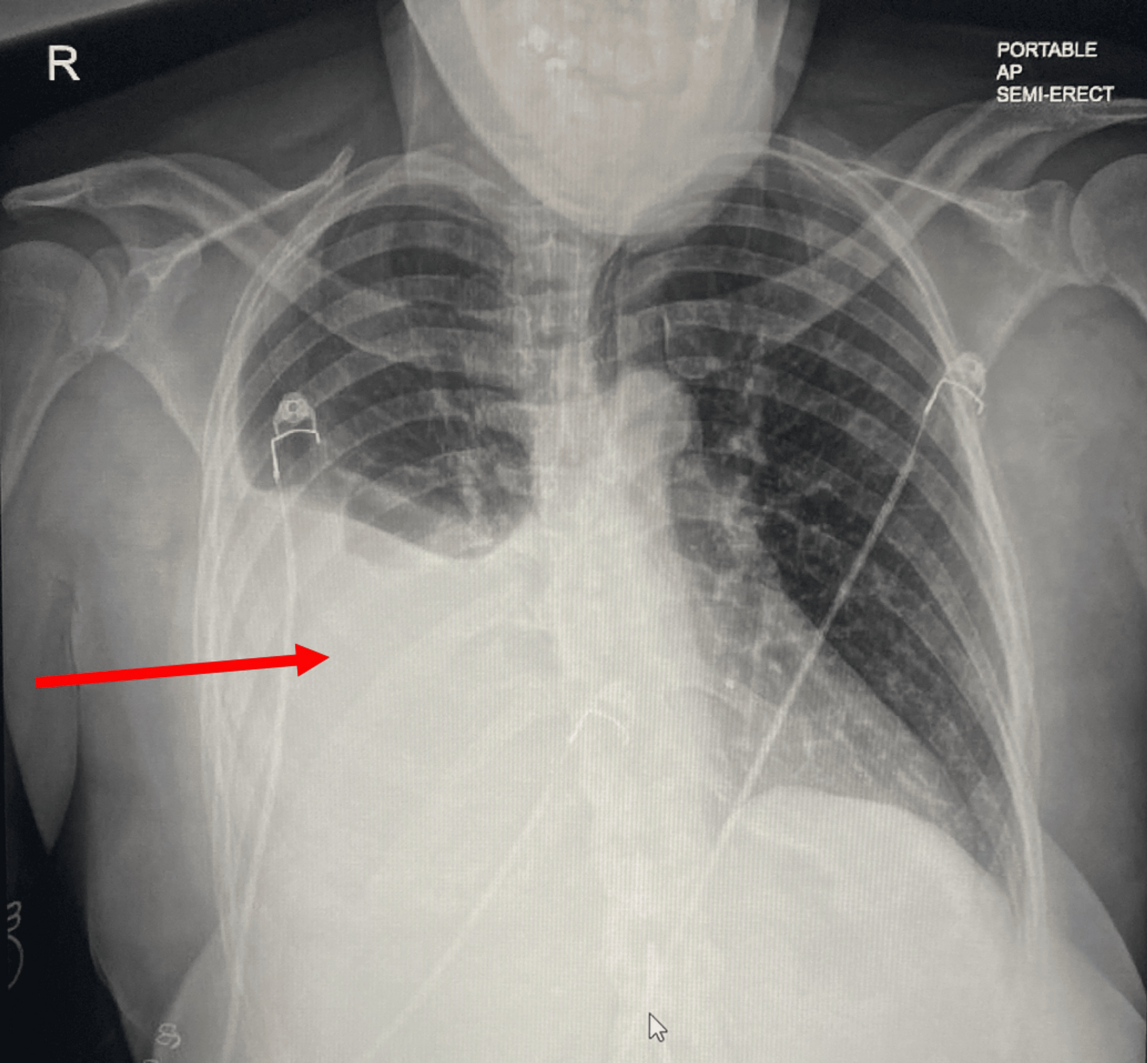
Chest Radiograph Showing a Large Right-Sided Pleural Effusion Before Thoracentesis Red arrow highlighting the area of interest.

**Figure 2:**
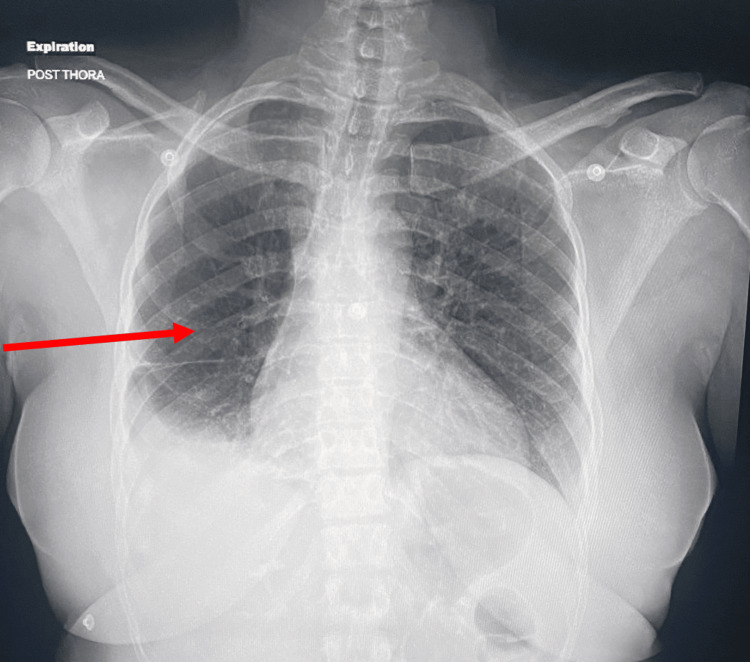
Chest Radiograph Showing a Significant Improvement in Right-Sided Pleural Effusion After Thoracentesis Red arrow highlighting the area of interest.

Chest radiograph revealed a large right pleural effusion with adjacent atelectasis/consolidation, prompting a computed tomography (CT) angiography of the chest for further evaluation, which confirmed the presence of a large right pleural effusion with complete collapse of the right lower lobe, partial collapse of the middle lobe, and a mild dependent atelectasis in the right upper lobe. 1000 mL of bloody fluid was aspirated on ultrasound-guided thoracentesis, and pleural fluid was sent for further investigation/cytology. The chest radiograph illustrates a large right-sided pleural effusion pre- and post-thoracentesis (Figures 1, 2).

Pleural studies were consistent with an exudative effusion: pleural fluid total protein: 5.6 g/dL, pleural fluid lactate dehydrogenase: 393 U/L, serum lactate dehydrogenase: 166U/L, and serum total protein: 8 g/dL. Laboratory values of the pleural fluid study are listed (Table 2).

**Table 2:** Laboratory Values of Pleural Fluid Study WBC: white blood cell, RBC: red blood cell, LDH: lactate dehydrogenase

Pleural Fluid Study	Laboratory Values	Reference Range
Pleural fluid color	Red	Colorless
Pleural fluid appearance	Clear	Clear
WBC pleural fluid	1034	<200 cells/uL
RBC pleural fluid	421000	<2000 cells/uL
Pleural fluid LDH	393	No reference range U/L
Pleural fluid total protein	5.6	No reference range g/dL

**Figure 3:**
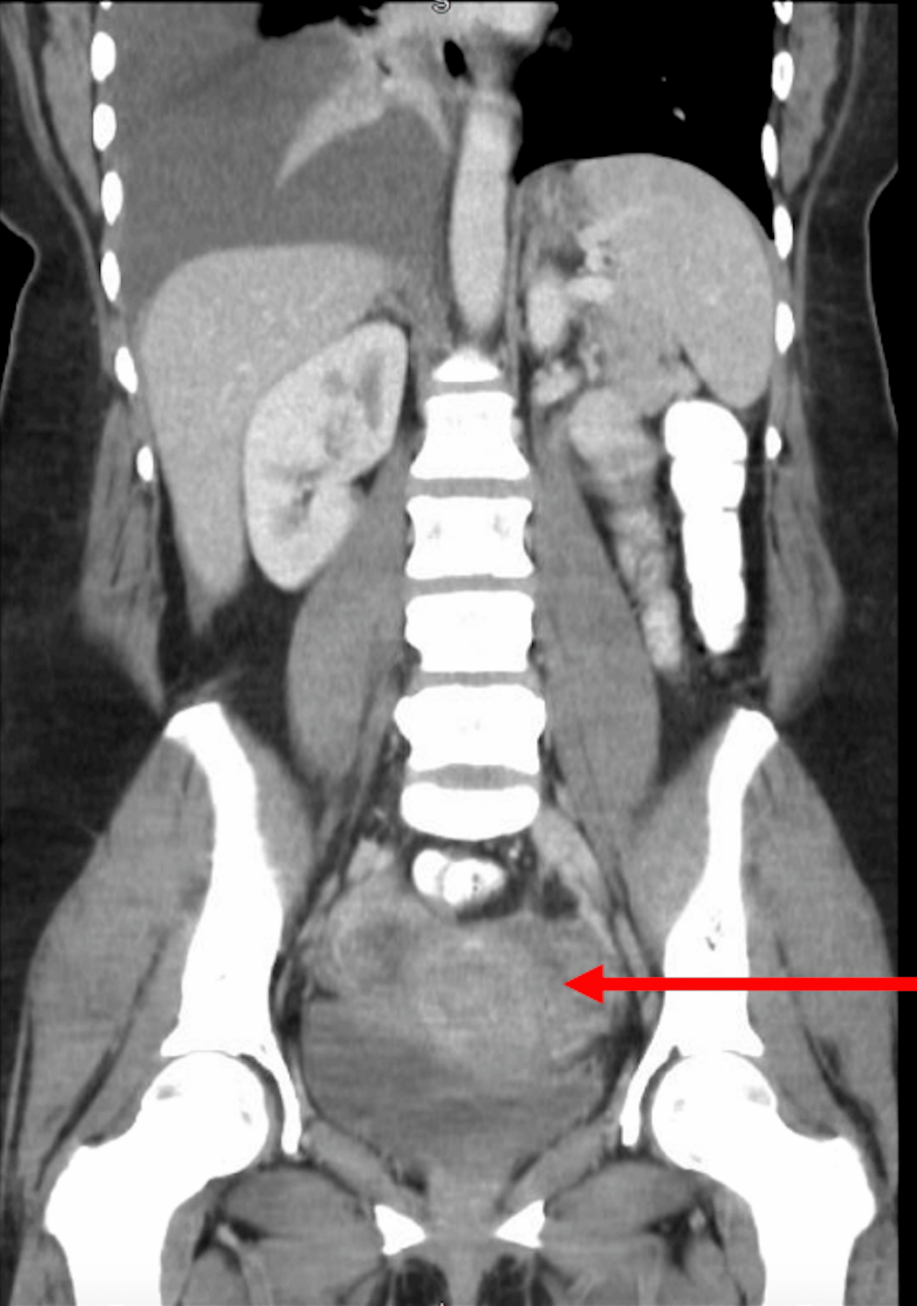
Coronal View of CT Abdomen/Pelvis Without Contrast Showing a Mixed Solid and Cystic Mass Within the Left Ovary Red arrow highlighting the area of interest. CT: computed tomography

Given the elevated cancer antigen (125) levels, CT abdomen/pelvis without contrast was obtained, which revealed a mixed solid and cystic mass measuring 6.7x2.5 cm within the left ovary/left adnexa, prominent right groin lymph nodes, and shotty mesenteric lymph nodes. CT abdomen/pelvis without contrast illustrates the suspicious mass in the left ovary (Figure 3).

During the hospitalization, she showed improvement in her symptoms, but rapid re-accumulation of fluid in the pleural space was observed; she underwent repeat thoracentesis with aspiration of 1500 mL of bloody fluid. Cytological results were suspicious for malignancy of ovarian origin at the time of discharge. Due to the high clinical concern of malignancy, she was referred for outpatient follow-up with gynecology/oncology. 

**Figure 4:**
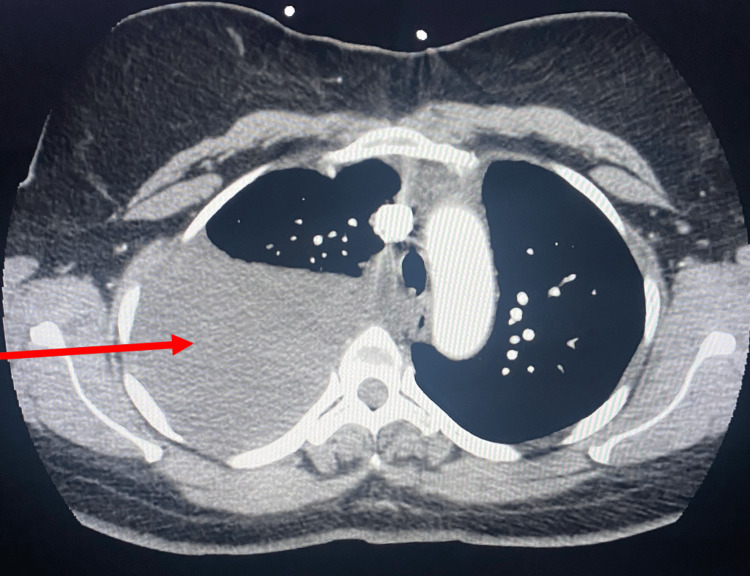
CT Angiography Chest Showing Re-accumulation of Large Right-Sided Pleural Effusion Red arrow highlighting the area of interest. CT: computed tomography

At the two-week follow-up after the procedure, she presented with complaints of shortness of breath on exertion and orthopnea ongoing since her discharge. A large right-sided pleural effusion occupying almost the entire right hemithorax with partial collapse of the right lung was seen on chest imaging. CT angiography chest shows a recurrent large right-sided pleural effusion (Figure 4).

**Figure 5:**
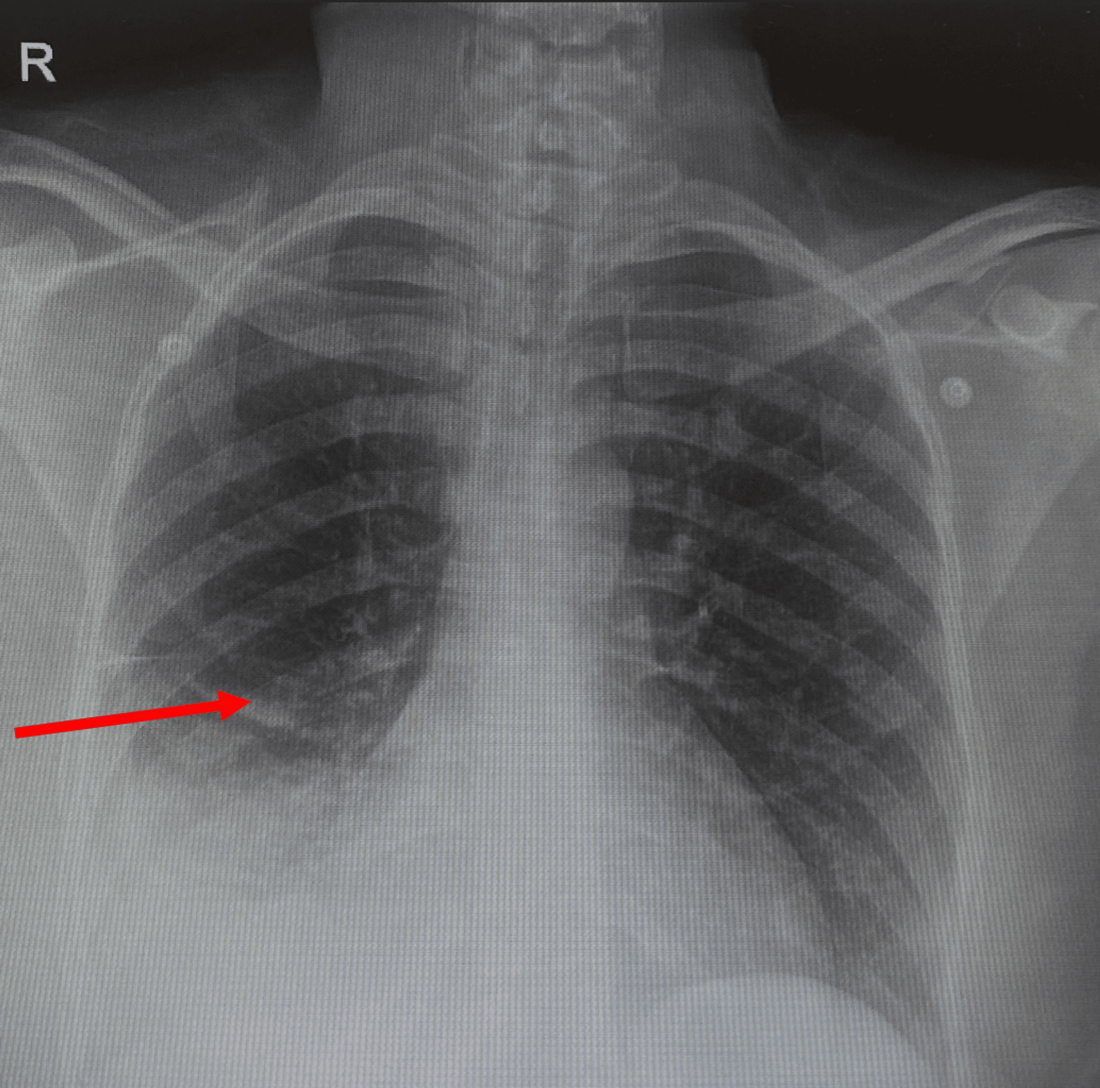
Chest Radiograph Showing Resolution of Recurrent Large Right-Sided Pleural Effusion After Chest Tube Insertion Red arrow highlighting the area of interest.

Pulmonary medicine was consulted, and she underwent a thoracentesis with aspiration of 1900 mL of bloody fluid; a chest tube was placed. Pleural fluid cytology was obtained, which showed hemosiderin-laden macrophages, lymphocytes with occasional eosinophils, and negative for malignant or atypical cells, no epithelial cells. A Chest x-ray was obtained after chest tube insertion, which showed significant improvement in effusion. The chest radiograph shows a significant improvement in the right-sided pleural effusion (Figure 5).

Given this concern, cardiothoracic surgery was consulted for evaluation, and she underwent video-assisted thoracoscopy with right robotic-assisted pleurectomy, pleurodesis, lymph node dissection, intercostal nerve blocks, and a pleural biopsy. Post-procedural chest imaging showed complete expansion of the lung. She was discharged home with a scheduled gynecology follow-up. Of note, the final pathology from her exploratory laparoscopy returned consistent with benign endometriosis. A re-review of her pathology at the initial presentation showed no overt evidence of malignancy in pleural cytology. 

At one week follow-up, she denied any dyspnea/orthopnea since the surgical procedure and was reportedly doing well. At six months follow-up, she was without any complaints. 

## Discussion

Endometriosis is the ectopic growth of endometrial glands and stroma outside the uterine cavity [4]. It can present early in the pubertal years at the onset of menarche, but prevalence is reported in 6-10% of women of reproductive age [4]. The classic symptom is "cyclic pain associated with the onset of menses." 

Many theories have been proposed regarding the pathogenesis of endometriosis; a similarity observed was based on these significant hallmarks: hormonal imbalance, genetic predisposition, and the concept of inflammation [4-6]. It remains a clinical diagnosis that requires proper history-taking and physical examination followed by diagnostic/therapeutic evaluation with laparoscopy, which has proven to be the gold standard based on a high clinical suspicion by the clinician [5].

Extra-pelvic/genital endometriosis is a rare presentation defined as the presence of ectopic endometrial glands/stroma outside of the pelvic cavity. Its prevalence has been observed in a slightly older population compared to the endometriosis age group. A few case reports have reported some of the following implantation sites: thoracic cavity, gastrointestinal tract, central nervous system, extremities, and skin [7]. 

Thoracic endometriosis, as the name implies, is defined as evidence of endometrial stroma/glands on histological specimens within the thoracic cavity. A few variants of its presentation have been described in the literature, including hemothorax, pneumothorax, hemopneumothorax, hemoptysis, and pulmonary nodules. Pneumothorax has been reported as the most common presentation of thoracic endometriosis [1,3,8,9].

A vital keyword, "catamenial," has been used frequently concerning thoracic endometriosis; it is defined as a "temporal relationship to the menstrual period." This unique characteristic is strongly linked to thoracic endometriosis and can be used to establish a diagnosis in most cases [1].

Hemothorax is the accumulation of blood in the pleural space, often caused by trauma. Less common causes include iatrogenic causes, spontaneous causes, neoplasm, anticoagulant use, endometriosis, rupture of pulmonary vascular malformations, and rupture of aneurysm thoracic arteries [2,10]. Thoracocentesis is the initial diagnostic approach followed by the evaluation of pleural fluid. In some literature, the use of prophylactic antibiotics within 24 hours of traumatic hemothorax drainage has been described, but no depth has been explored regarding its use in spontaneous hemothorax [10].

The typical presentation of hemothorax associated with thoracic endometriosis is usually a recurrent right-sided effusion, and the chief complaint is shortness of breath in the presence/absence of chest pain. Without a high clinical suspicion, this diagnosis is quickly dismissed from the differential diagnoses. Most patients tend to have recurrent symptoms before a multidisciplinary approach is undergone to reach the diagnosis, including a team of cardiothoracic surgeons, pulmonologists, and gynecologists [11,12].

A few theories have been proposed to explain the cause of thoracic endometriosis. The most widely acceptable theory is Sampson's theory, which argues that endometrial cells reach the peritoneal/pleural cavity via the fallopian tubes through a process termed "retrograde menstruation." The predominance of right-sided thoracic involvement strongly supports this theory [1,12].

Other theories, such as the lymphatic/hematogenous dissemination theory, coelomic metaplasia theory, stem cell theory, and the prostaglandin theory, propose that dissemination of endometrial cells via lymphatic or hematogenous routes, metaplasia of coelomic epithelial cells, endometrium/bone marrow stem cells as the precursors for ectopic endometrial implants, and constriction of bronchioles and blood vessels resulting in subpleural blebs/bullae rupture because of increased prostaglandin circulation during menstruation could be the culprit behind the pathogenesis [1,3,13].

In summary, like pelvic endometriosis, the etiology of thoracic endometriosis is suspected to be multifactorial, as not one single theory can thoroughly explain its clinical presentation. 

A readily accessible form of imaging during the initial presentation is a chest radiograph. CT scans can also be utilized depending on their availability. A diagnostic dilemma is still encountered given the temporal relationship to menses; a solution might be to obtain imaging at the time of presentation and compare it to imaging obtained during menses/mid-cycle [8,12]. Diagnostic modalities such as pleural fluid studies/evaluation, bronchoscopy, cytology, and biopsy can be helpful, but video laparoscopy and video-assisted thoracoscopic surgery remain the gold standard for diagnosis/treatment. Although not possible in all cases of thoracic endometriosis, the presence of endometrial glands/stroma on histopathology provides a confirmatory and definitive diagnosis. A few pieces of literature report using CA-125 levels as a guide for diagnosis, but this needs to be more specific and could be misleading for malignancy, as in our case report. 

A multidisciplinary approach is required to achieve adequate management of thoracic endometriosis. At the presentation, symptomatic management is indicated with thoracocentesis, oxygen supplementation, and as-needed pain medication. Similar to the management of pelvic endometriosis, hormonal therapy appears to be the first line for thoracic endometriosis; gonadotropin-releasing hormone analogs and oral contraceptives can be used for this purpose. Definitive management of thoracic endometriosis can be achieved by utilizing both hormonal therapy and surgical therapy. Based on the available literature, neither treatment has proven superior, as either therapy alone has proven unsuccessful in preventing symptom recurrence. In instances where recurrence occurs even after surgical treatment, pleurodesis can be utilized to avoid future recurrence. Hormonal therapy should be continued for six to 12 months following surgical treatment. Some patients subsequently undergo a total hysterectomy and bilateral salpingo-oophorectomy to exclude the possibility of malignancy; this should also be considered strongly in older patients who no longer desire fertility [12].

## Conclusions

The diagnosis of this patient posed a dilemma as there was initial suspicion of ovarian cancer and Meig's syndrome, which can have similar presentations. Thoracic endometriosis relies on high suspicion by clinicians, although this may be difficult on the initial encounter as the diagnosis is often delayed, leading to further complications and recurrent hospitalizations. Despite this barrier, it should remain on the differential diagnosis, and further investigation should be performed in females of childbearing age who present with an unexplained cause of shortness of breath accompanied by a pleural effusion on chest imaging. Surgical intervention is always often necessary, and postoperative hormonal suppression can prevent relapse. 
